# Experience of Emergency Peripartum Hysterectomies at a Tertiary Care Hospital in Quetta, Pakistan

**DOI:** 10.5402/2011/854202

**Published:** 2011-09-29

**Authors:** Mahrukh Fatima, Pashtoon Murtaza Kasi, Shahnaz Naseer Baloch, Abaseen Khan Afghan

**Affiliations:** Department of Obstetrics and Gynecology, Bolan Medical College, 8-13/36 Kasi Road, Quetta, Balochistan 87300, Pakistan

## Abstract

Emergency peripartum hysterectomy (EPH) is associated with significant morbidity and mortality worldwide. The purpose of our paper was to determine the incidence, morbidity, and mortality of EPH done at our institution; the largest tertiary care government hospital in the city of Quetta, Pakistan. During the study period there were 12,642 deliveries, out of which 46 women had undergone an EPH, translating into an incidence of *∼*4 per 1,000 births. Disturbingly, 82.6% of these patients had received no antenatal care prior to their presentation. There were 4 (8.7%) maternal deaths and 31 (67.4%) perinatal deaths. The commonest indication noted was uterine rupture in 21 (45.7%) cases. Lack of antenatal care is indeed a modifiable factor that needs to be addressed to help reduce maternal and fetal morbidity/mortality not only from emergency hysterectomies but also from all other preventable causes.

## 1. Introduction

Emergency peripartum hysterectomy (EPH) is associated with significant morbidity and mortality worldwide. They are seen more often in developing countries due to decreased availability and lack of uptake of antenatal care services especially in the rural areas. There also appears to be a rise of EPH in the developing world as well [1].

The purpose of our study was to review the emergency peripartum hysterectomies (EPH) done at our institution. Our specific aims were then to determine the incidence, the associated morbidity and mortality, risk factors, and complications noted at our institution. This would help highlight the lack of availability and utilization of antenatal services, identify avoidable factors, and stress the need to organize health care services so as to improve maternal and fetal outcome.

## 2. Materials and Methods

Ethical approval for the study was obtained from the Department of Obstetrics and Gynecology, Bolan Medical College, Quetta, Pakistan, and the research conducted was performed according to the Declaration of Helsinki. 

The study was carried out at the largest tertiary care government hospital in the city of Quetta in Balochistan, the largest province in Pakistan. Quetta is a metropolitan city and the capital of the province. People belonging to different castes live here along with many refugees who were from the adjacent wartorn country of Afghanistan and migrated during the early 1980s and 1990s. This represents one of the major teaching/tertiary care centers for the province. 

Initial part of this work was done and data collected prospectively from 1994 as part of the FCPS dissertation of author M. Fatimathr. Due to lack of complete medical records, we were able to obtain data for all cases that needed EPH in Gynecology Unit I and II at Sandeman Medical College Hospital, Quetta, Pakistan over a period of 2 years from September 25th 1994 to September 1st 1996. Records after transfer of the gynecology department to another institution were not available for review. 

During this period there were 12,642 deliveries, out of which 11,960 were vaginal deliveries and 682 caesarean sections. EPH was defined as one performed after 20 weeks gestation for uncontrollable uterine bleeding not responsive to conservative measures occurring at any time before and after delivery but within the first 6 weeks postpartum. During the study period, 46 women were noted to have undergone an emergency peripartum hysterectomy. Data regarding their basic demographics, mode of delivery, maternal and fetal outcome, along with associated complications was then collected and entered into a database developed in Microsoft Access 2000. This was then imported into the Statistical Package for Social Sciences version 14.0 (SPSS Inc., Chicago, IL, USA) for further analysis.

## 3. Results and Discussion

As noted above, 46 women were identified who underwent an emergency peripartum hysterectomy from a total of 12,642 deliveries. This translates into an incidence of *∼*4 per 1,000 births. Compared to a study done in Australia where the incidence was 0.85 per 1000 births, this represents a rate which is approximately 5-fold higher [1]. In their study, only 33 EPH were noted among 33,998 births over a 10-year period whereas in another study in Turkey, 34 cases were identified over a 10-year period [2]. Similarly, in a study from a community teaching hospital in New York, 48 cases were noted over an 8-year period; translating to an incidence of 1.4 per 1000 births [3]. Compared to the aforementioned numbers, our study just over a 2-year period at a tertiary care facility serving one of the least developed parts of Pakistan represents alarmingly high numbers. 

Table 1 outlines the basic demographic data regarding this subset of patients. Disturbingly, 82.6% of these patients had received no antenatal care prior to their presentation. The majority of these patients lived in rural areas with the monthly income of most of them being less than $60. 

Review of the morbidity and mortality data is outlined in Table 2. Unfortunately, this is also associated with poor fetal outcomes as outlined in Table 3. 

There were 4 (8.7%) maternal deaths. One patient died due to hemorrhagic shock from a ruptured uterus, and 3 patients died due to sepsis after being referred from periphery of the city after obstructed labor. Compared to a study in Ghana, “there were no maternal deaths but there were 7 near-missed fatalities”; whereas a study in Maryland noted 2 deaths in 34 patients from 1991 to 2001 [4, 5]. A study done at another tertiary care facility in another city of Pakistan noted maternal mortality in 4 (19%) of their cases [6]. 

The hospital stay of these patients ranged from 8 to 32 days, with a mean of 16.5 days. 20 (43.5%) patients stayed for 10 days or less, 23 (50%) for 11–20 days, and 3 patients stayed for 30–32 days. This represents significant healthcare-associated costs for patients in a country where they often end up bearing the bulk of the costs, with significant social and economic consequences. 

Among 46 cases of peripartum hysterectomies the range of parity was from 0–15. Only one patient was primigravida, who required hysterectomy due to placenta percreta, two were second gravid both requiring hysterectomies due to rupture of a previous caesarean scar; while the rest of the patients were multiparous. About 70% of patients were grand multipara (5 or more previous deliveries); this was also noted by Imudia et al. where high parity remained a risk factor for complications in this subset of patients [7]. Likewise, in a study from Brigham and Women's Hospital, Boston, MA, the “incidence of EPH increased from one in 143 deliveries in women with one prior live birth and a prior cesarean section to one in 14 deliveries in multiparous women with four or more deliveries with a history of a prior cesarean section” [8]. 

42/46 patients had total abdominal hysterectomy performed; 4 had subtotal hysterectomies. Even though the numbers are small, subtotal hysterectomy is also a reasonable alternative in emergency obstetric hysterectomy [9].

The majority of the complications noted in these patients were infectious complications (fever, wound site infection, and urinary tract infection) followed by complications related to the emergent surgery itself. This is similar to the study done in New York where postoperative febrile morbidity constituted 34% of the cases [3]. 

Almost all the cases were done in an emergent setting (44/46; 95.6%); only 2 cases were done in an elective fashion. This is also similar to a study done in Detroit, where 14/158 cases were done in an elective fashion whereas the remainder 144 had to be performed emergently due to complications encountered at cesarean section [7].

There were 31 (67.4%) perinatal deaths noted at our institution. Twenty-nine were stillborn, 21 of which were due to a ruptured uterus and 5 were due to abruption placentae, 3 were due to uterine infection, and 2 were neonatal deaths due to aspiration pneumonia. These, as noted before, are very disturbing numbers. In a study from Vienna, the newborns of these women had a lower birth weight, significantly lower APGAR scores at 1 and 5 minutes, and were more often transferred to the neonatal intensive care unit (NICU) [10]. However, the disturbingly high neonatal mortality numbers here are a possible reflection of delayed presentation of these patients with no prior antenatal care after having had instrumentation done at homes or by local untrained birth attendants. 

All the patients were noted to have received blood transfusion during or in the immediate postoperative period where indicated. This is also similar to the study done by Awan et al. in Australia where also all patients required blood transfusions [1].

In our series of patients, the commonest indication for an EPH was uterine rupture in 21 (45.7%) cases. In 10 (47.62) cases the etiological factors appeared to be related to presentation of multipara initially to traditional birth attendants (TBAs) in rural peripheral areas or at home. In 4 cases obstructed labor due to malpresentation and cephalopelvic disproportion neglected by TBAs (or “Dai”, local untrained birth attendant) appeared to be associated. One patient (4.76%) had traumatic rupture of uterus during peripartum manipulation at an outside hospital. 4 patients had rupture of a previous caesarean section scar. Two patients of lower segment caesarean section were tried at home by TBAs. Patients who had rupture of unscarred uterus were all grand multipara and had a history of presenting initially to a local untrained birth attendants at home prior to coming to the hospital.

In the remaining patients EPH was due to postpartum hemorrhage (PPH); 10 (21.74%) cases had PPH due to uterine atony. Less frequent indications were abruption 5 (10.87%) covualaire uterus, placenta previa 2 (4.35%), and placenta percreta 2 (4.35%). This is similar to another study from Hyderabad, Pakistan, where also the main indication for EPH was rupture of the uterus 7 (33.3%) [6].

The etiologies noted in our subset of patients are different from the developed world where abnormal placentation resulting in hemorrhage was the most common cause. In a study done in The Netherlands, the main indication for EPH was placenta accreta (50%), followed by uterine atony (27%) [11]. Similarly, even in a small study of 17 patients in Saudi Arabia and 54 patients in Republic of Korea, uterine atony appeared to be the most common cause [12, 13]. In a study from Turkey, uterine rupture was noted to be the cause in 21% of the cases; there too, uterine atony was responsible for more than 42% of the cases [14]. In our cases, instrumentation and possible neglect during the births at outside peripheral hospitals and/or homes under untrained birth attendants appears to be associated with the patients presenting with uterine rupture.

## 4. Conclusions

- In our study, we have identified not only the incidence of emergency peripartum hysterectomy (EPH) but also highlighted the significant morbidity, mortality, and associated healthcare costs.
- Lack of antenatal care, which appears to be a common theme, is indeed a modifiable factor that needs to be addressed to help reduce maternal and fetal morbidity/mortality not only from emergency hysterectomies but also from all other causes which appear to be associated with lack of antenatal care services.
- In developing countries like Pakistan, maternal and neonatal mortality still represent a significant burden, especially in underdeveloped areas like the city of Quetta, Pakistan.
- More attention to this would need to be given by the government and the health departments of the country for interventions to not only provide healthcare services but also increase uptake of these services in rural areas of Pakistan, where still a significant proportion of deliveries are performed under the supervision of untrained birth attendants.
- At the same time, in developing countries, EPH is almost always an emergency with high risk for significant blood loss. “The obstetrician should be ready to do it, and an early decision should save blood and prevent complications. Postoperative complications, mostly bleeding and infections may be severe. Early intervention and proper technique facilitate good outcomes [15].”
- Since most of these cases are associated with prior Cesarean section, as noted by Daskalakis et al., “every attempt should be made to reduce the cesarean section rate by performing this procedure only for valid clinical indications [16].”

## Figures and Tables

**Table 1:** Basic demographics and relationship of peripartum hysterectomy with parity.

	Number of cases	%
(1) Peripartum hysterectomy	46	0.36 (*∼*4 per 1,000 births)
(2) Mode of delivery:		
Vaginal	1	2.2%
Caesarean	45	97.8%
(3) Monthly income (Rupees)		
<5,000 (*∼*$60)	38	82.6%
5,000–10,000 ($60–120)	5	10.9%
>10,000 ($120)	3	6.5%
(4) Area of residence		
Rural	32	69.6%
Urban	14	30.4%
(5) Antenatal care visit Received prior to presentation		
Yes	8	17.4%
No	38	82.6%
(6) Relationship with maternal age		
20–24	2	4.3%
25–30	10	21.7%
31–35	22	47.8%
36 and above	12	26.0%
(7) Relationship with parity		
Primigravida	1	2.2%
1–4	13	28.3%
5–15	32	69.6%
(8) Duration of pregnancy		
Full term	38	82.6%
Preterm	2	4.3%
Postterm	6	13.1%

**Table 2:** Maternal morbidity and mortality in patients with emergent peripartum hysterectomy.

Variable	*N*	%
(1) Maternal mortality	4	8.7%
(2) Mean hospital stay	16.4 days	
(3) Mean duration of surgery	120 minutes	
(4) Prolonged hospital stay (>14 days)	20	43.5%
(5) Mean units of packed red blood cells transfused	3.1 units	
(6) Total number of packed red blood cell transfusions (during entire hospital stay)		
1–3 units	33	71.8%
4–6 units	11	23.9%
7 units	2	4.3%
(7) Complications		
Fever	20	43.5%
Wound infection	15	32.6%
UTI	10	21.8%
Vesicovaginal fistula	6	13.0%
Ileus	6	13.0%
Transfusion reaction	5	10.9%
Sepsis	5	10.9%
Prolonged intubation	2	4.4%
Ureteral/bladder injury	2	4.4%
Pneumonia	1	2.2%
DVT	1	2.2%

**Table 3:** Fetal and neonatal outcomes.

(1) Fetal outcome		
Stillbirth	29	63%
Live born	17	37%
(2) Neonatal outcome of 17 Liveborn		
Neonatal deaths	2	
Alive	15	
(3) Causes of stillbirth		
Ruptured uterus	21	72.4%
Abruptio placentae	5	17.2%
Uterine infection/sepsis	3	10.4%
